# Effectiveness of insecticide-impregnated dog collars in reducing incidence rate of canine visceral leishmaniasis: A systematic review and meta-analysis

**DOI:** 10.1371/journal.pone.0238601

**Published:** 2020-09-03

**Authors:** Yonas Yimam, Mehdi Mohebali

**Affiliations:** 1 Department of Medical Parasitology and Mycology, School of Public Health, Tehran University of Medical Sciences, Tehran, Iran; 2 Department of Biology, Faculty of Natural and Computational Sciences, Woldia University, Woldia, Ethiopia; 3 Centers for Research of Endemic Parasites of Iran (CREPI), Tehran University of Medical Sciences, Tehran, Iran; Instituto Oswaldo Cruz, BRAZIL

## Abstract

Canines are proven reservoir hosts of *Leishmania infantum*, the causative agent of human zoonotic visceral leishmaniasis, and therefore domestic dogs play a central role in transmitting the disease to humans. Studies on the effect of insecticide-impregnated dog collars for controlling canine visceral leishmaniasis (CanL) have been increasing; however, meta-analysis has not been conducted. This study assessed the effectiveness of insecticide-impregnated dog collars for preventing CanL. We searched (PubMed, Web of Science, Scopus, Embase, Ovid Medline(R), and Cochrane library), from inception until 2 May 2020. Two authors independently performed articles screening and data extraction. We applied the RoB 2.0 tool to evaluate the risk of bias in randomized trials, while the ROBINS-I tool was used for non-randomized trials. I-squared statistics(I^2^) and funnel plot and Egger’s test, respectively, were used to assesses heterogeneity between studies and publication bias. Relative Risk (RR) and 95% Confidence Interval (CI) were calculated using the random-effects model in Stata 14 software. Out of 242 citations identified, 14 studies comprising 3786 collared dogs and 3428 uncollared dogs were eligible for meta-analysis. The use of deltamethrin-impregnated dog collars(DMC) showed an overall effectiveness of 54% (95%CI: 35–65%, I^2^ = 63.2%, *P* = 0.002) in decreasing incidence of CanL, while 10% imidacloprid and 4.5% flumethrin collars provided an overall effectiveness of 90% (95%CI: 80–96%, I^2^ = 0.0%, *P* = 0.376). DMC efficacy stratified by follow-up duration was estimated to be 58% (RR = 0.42, 95%CI: 0.20–0.87), 54% (RR = 0.46, 95%CI: 0.31–0.68), 53% (RR = 0.47, 95%CI: 0.29–0.82) for follow-up periods of 5 to 6 months, 1 year and 2 years, respectively. The current evidence indicates that using insecticide-impregnated dog collars can reduce the risk of CanL caused by *L*. *infantum*. Therefore, insecticide-impregnated dog collars could be a viable alternative for inclusion as a public health measure for controlling CanL.

## Introduction

Canine leishmaniosis (CanL), arthropod-borne parasitic zoonosis, caused by *Leishmania infantum*, mostly occur in the Mediterranean region, and South America [1, 2]. CanL affects at least 2.5 million dogs in the Mediterranean region, and the outcome of infection may be asymptomatic or may evolve into a potentially fatal viscerocutaneous disease [2, 3]. CanL infected dogs, both symptomatic and asymptomatic dogs, are important reservoirs for zoonotic visceral leishmaniasis and thus facilitate the transmission of *L*. *infantum* to other dogs and humans [4, 5]. In areas where human zoonotic visceral leishmaniasis is endemic, implementation of dog-focused control measures can be of dual importance in protecting dogs from the suffering of the disease and mitigating the high risk of disease transmission to humans [6, 7].

Over the past two decades, numerous CanL control tools have been developed, such as insecticides, chemotherapy, and vaccine; however, from the public health perspective, insecticide-impregnated collars are the recommended tool for better CanL control [8–11]. Antileishmanial treatments of dogs’ lead to clinical improvement of treated dogs and decrease dogs’ infectiousness, limiting the transmission of the parasite from canines to phlebotomine sand flies; However, owing to drugs limitation in the parasitological cure, complete parasite clearance is not frequently achieved and dogs may remain infectious to sand flies. And this leads to recurrences of the disease in dogs, and it requires continuous treatment, which is not cost-effective and practically unfeasible from a public health perspective [4, 12]. In recent years, the use of CanL vaccines has shown a promising result in keeping the parasite burden low and improving dogs’ clinical signs, but current CanL vaccines do not protect the establishment of infection. Though there have been substantial efforts in the development of a robust and effective vaccine, insecticides(pyrethroids) in the form of insecticide-impregnated dog collars have been used to prevent sand fly bite, and consequently spread of the disease [6, 8, 9].

Thus far, there are two proven insecticide-impregnated dog collars; deltamethrin-impregnated collar and flumethrin and imidacloprid containing collars (flumethrin 4.5%, imidacloprid 10%), which have both anti feeding and insecticidal effects on the sand fly, and subsequently reduce infection in dogs and humans [13–15]. Also, scientific studies on the effectiveness of insecticide-impregnated dog collars for CanL control are growing, and studies have shown that the use of insecticide-impregnated collars can reduce the incidence of CanL despite protection rate are heterogeneous [16]. In 2014, a systematic review was conducted on the efficacy of insecticide in the control of *L*. *infantum* in dogs [17]; but, this systematic review does not include meta-analysis and recently licensed insecticide-impregnated collar (10% imidacloprid and 4.5% flumethrin). And after the previous systematic review, additional studies have conducted. We, therefore, undertaken this updated systematic review with meta-analysis to provide insights on the overall effectiveness of insecticide-impregnated dog collars in reducing CanL, thereby providing more evidence for guiding intervention policy.

## Methods

### Design

The present systematic review with a subsequent meta-analysis was carried out in compliance with the guidelines for systematic reviews and meta-analyses (PRISMA) (S1 Checklist) to describe the results and PICOT strategy (P-Population, I-Intervention, C-Comparison, O-Outcome and T-Follow-up time/Type of study) applied to structure the search and subsequently for selection criteria of studies. In particular, P: Dogs(canines), I: insecticide-impregnated dog collars (deltamethrin or 10% imidacloprid and 4.5% flumethrin), C: dogs that are not fitted with insecticide-impregnated dog collars. O: post-intervention incidence of CanL and follow-up period, T: type of study: we included both randomized and non-randomized trials and T: Follow-up time: we included studies that have five or more months follow-up duration.

### Data sources, search strategy and study selection

We systematically searched PubMed, Web of Science, Scopus, Embase, Ovid Medline(R), and Cochrane Library from inception until 2 May 2020. We also attempted to include additional potentially relevant studies by applying backward search (snowballing) of bibliographies of selected studies and five relevant previous systematic reviews and forward search (citation tracking) in google scholar. For the systematic search of articles, we applied Medical Subject Headings (MeSH), Embase subject headings (Emtree), and keywords. We used the following search terms; "canine leishmaniasis", "canine leishmaniosis", "canine visceral leishmaniasis", "zoonotic visceral leishmaniasis", "dog collars", "4% deltamethrin", "deltamethrin", "insecticide-impregnated dog collars", "deltamethrin-impregnated dog collars", "10% imidacloprid and 4.5% flumethrin", and "Flumethrin-Imidacloprid Collar" alone or in combination using a Boolean operators like AND and/or OR. We restricted our search to studies published in the English language. (S1 File).

Two authors independently performed the literature search, screenings of searched citations, and quality assessments of eligible studies. All citations searched were exported to Endnote 8 and screened in three steps. First, we removed duplicate citations; second, titles and abstracts were critically reviewed; Third, potentially relevant titles and abstracts were further screened for their full-texts. Any differences between the two authors were resolved by discussion and consensus.

### Eligibility criteria and data extraction

Studies were considered appropriate for inclusion if they fulfill the following criteria: (1) original peer-reviewed studies (randomized interventional studies and non-randomized interventional studies); (2) studies that diagnose CanL using serological or parasitological or molecular methods alone or studies that used a combination of those methods; (3) comparison arm contained uncollared dogs; (4) studies that measure the effectiveness of insecticide-impregnated dog collars on the incidence of CanL; (5) studies published in the English language. We excluded studies with the following criteria: non-original studies, studies that did not have comparator (control group), studies that do not measure the effectiveness of insecticide-impregnated dog collars, and studies not published in the English language.

Data concerning the effectiveness of insecticide-impregnated dog collars in reducing CanL were extracted using a pre-prepared data extraction format. Data regarding the following information were captured: (1) author/s name and year of publications; (2) study location; (3) study design; (4) types of intervention; (5) diagnosis method/s; (6) measure of effect; and (7) follow up duration.

### Risk of bias assessment

To assess the risk of bias in randomized interventional studies, we used the following five domains provided by the RoB 2.0 tool: (1) bias arising from the randomization process, (2) bias due to deviations from intended interventions, (3) bias due to missing outcome data, (4) bias in the measurement of the outcome and (5) bias in the selection of the reported result [18]. To assess the risk of bias in non-randomized interventional studies, we used the ROBINS-I tool. We evaluated the following domains using the ROBINS-I tool: (1) bias due to confounding, (2) bias in the selection of participants into the study, (3) bias in classification of interventions, (4) bias due to deviations from intended interventions, (5) bias due to missing data, (6) bias in the measurement of the outcome and (7) bias in the selection of the reported result [19].

### Data analysis

We implemented *metan* command in Stata version 14 statistical software for analysis. Review Manager (RevMan, version 5.3) was used to show the risk of bias summary. We used 2 x 2 tables to extract data from all eligible studies. When there was no CanL positive dog (zero cases) in the treatment group, we added 0.5 contingency correction to each cell. The pooled RR and the corresponding 95% CI were calculated using the DerSimonian-Laird method for the random-effects model based on the inverse variance approach for measuring weight. We calculated RR as follows: RR = incidence of CanL on collared dogs (intervention group)/incidence of CanL on uncollared dogs (control group). The effectiveness (%) of insecticide-impregnated dog collars in protecting against CanL was calculated as follows: effectiveness (%) = (% of positive dogs in the control group—% of intervention group)/ (% of positive dogs in the control group) ×100. To assess potential sources of heterogeneity, we carried out subgroup analysis stratified by diagnostic methods, follow-up duration, and sensitivity analysis; but, due to an insufficient number of studies, we did not investigate the impact of risk of bias on a pooled estimate. I^2^ statistics was used to explore heterogeneity between studies. The percentage of I^2^ was determined using the formula of Higgins and Thompson as [(Q−df)/Q]×100% where df is degrees of freedom (number of studies minus 1) [20]. I^2^ values of <30%, 30–75%, and <75% were deemed as low, moderate, and high heterogeneity, respectively, and the P-value was set at 0.05. We assessed publication bias qualitatively and then quantitatively confirmed using the funnel plot and Egger's test for small-study effects, respectively. Since publication bias was detected, we applied Duval and Tweedie's non-parametric/ trim and fill method to adjust the pooled estimate. We also carried out one study-leave-out sensitivity analysis to investigate the effect of each study on the pooled effect size estimate.

## Results

**Fig 1:**
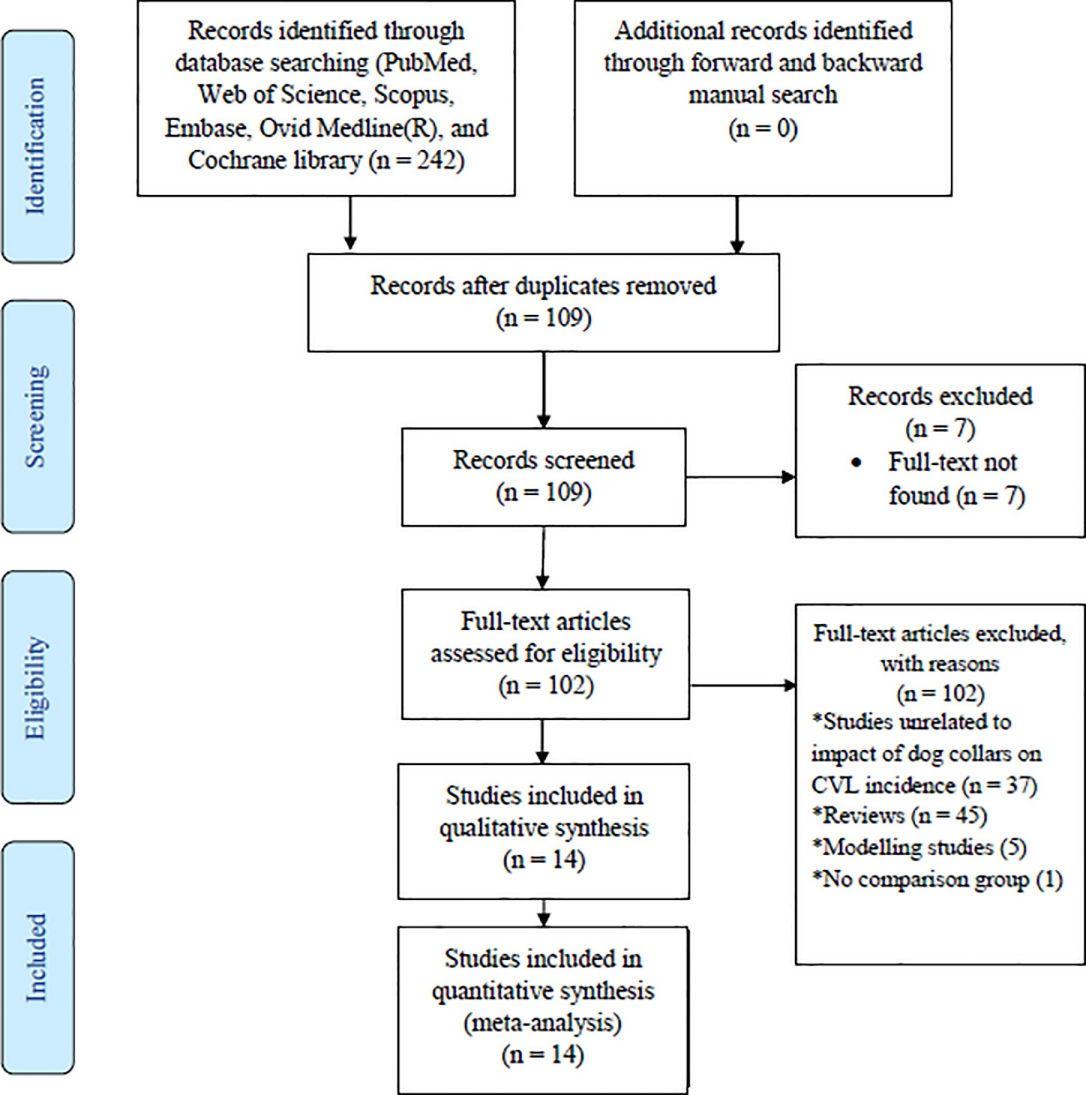
PRISMA flowchart for selecting eligible studies on the effectiveness of insecticide-impregnated dog collars for reducing the incidence of canine visceral leishmaniasis.

The process of articles search, screening, selection of eligible studies for this study is presented in Fig 1. We identified a total of 242 potentially relevant articles from six electronic databases, 133 of which were excluded as duplicates, and 109 articles were screened for titles and abstracts. The references of all 109 studies screened are provided in supporting information (S2 File). Three citations were excluded due to the absence of their abstracts. With the reason that their full-texts were not identified, four abstracts were excluded, out of which 2 studies are reviews, 1 study is unrelated to our outcome of interest, and 1 study evaluated the effect of DMC in reducing CanL incidence. After de-duplication and titles and abstracts screening, 102 full-text papers were critically assessed. A total of 88 full-text articles were excluded for the reasons that they didn’t meet pre-established inclusion criteria. Finally, a total of 14 studies included for the meta-analysis.

### Characteristics of selected studies and their quality assessment

**Fig 2:**
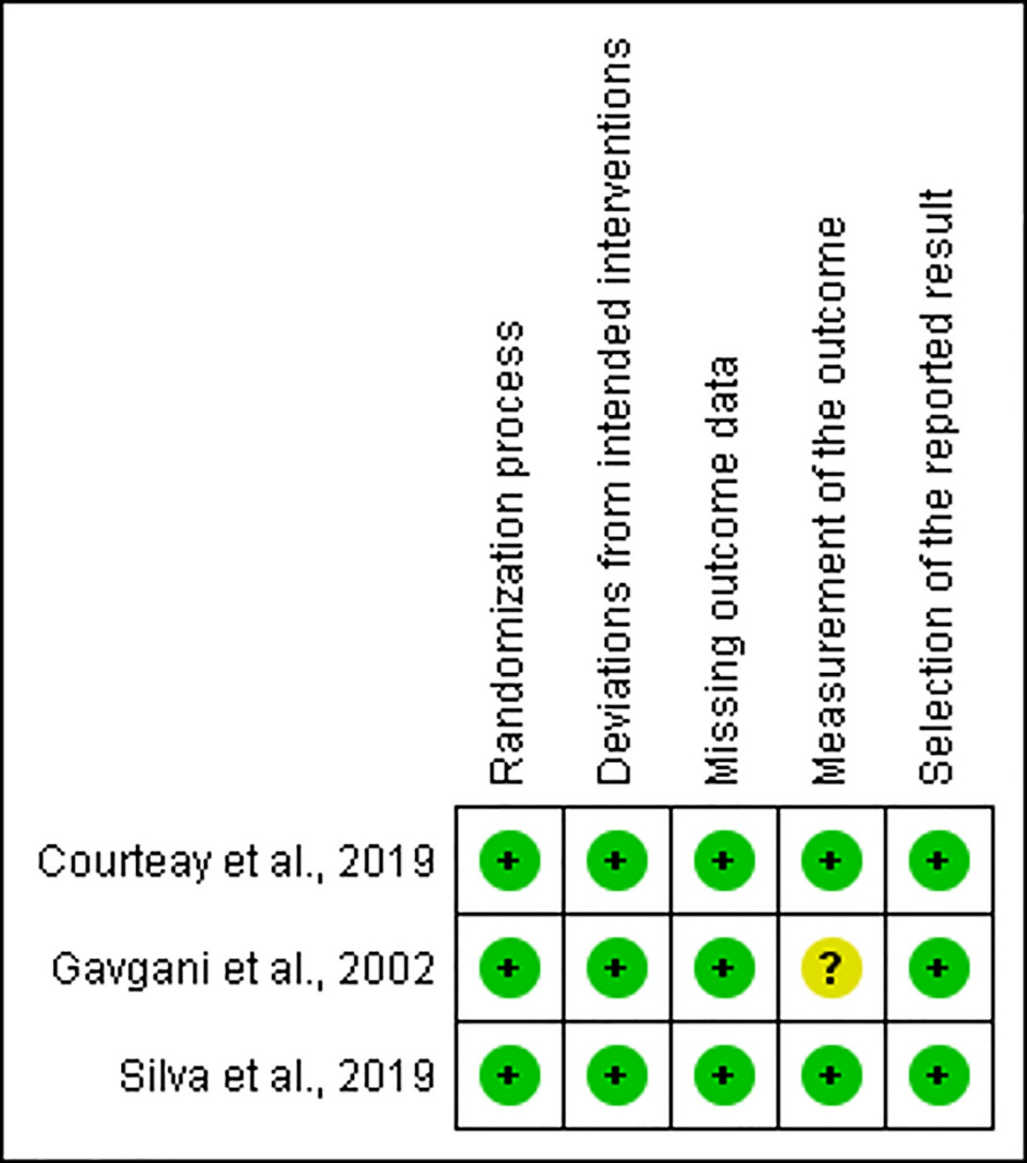
Summary of risk of bias assessment for randomized controlled intervention studies using ROB.2 tool. "+": low risk of bias; "?": unclear risk of bias; "−": high risk of bias.

**Fig 3:**
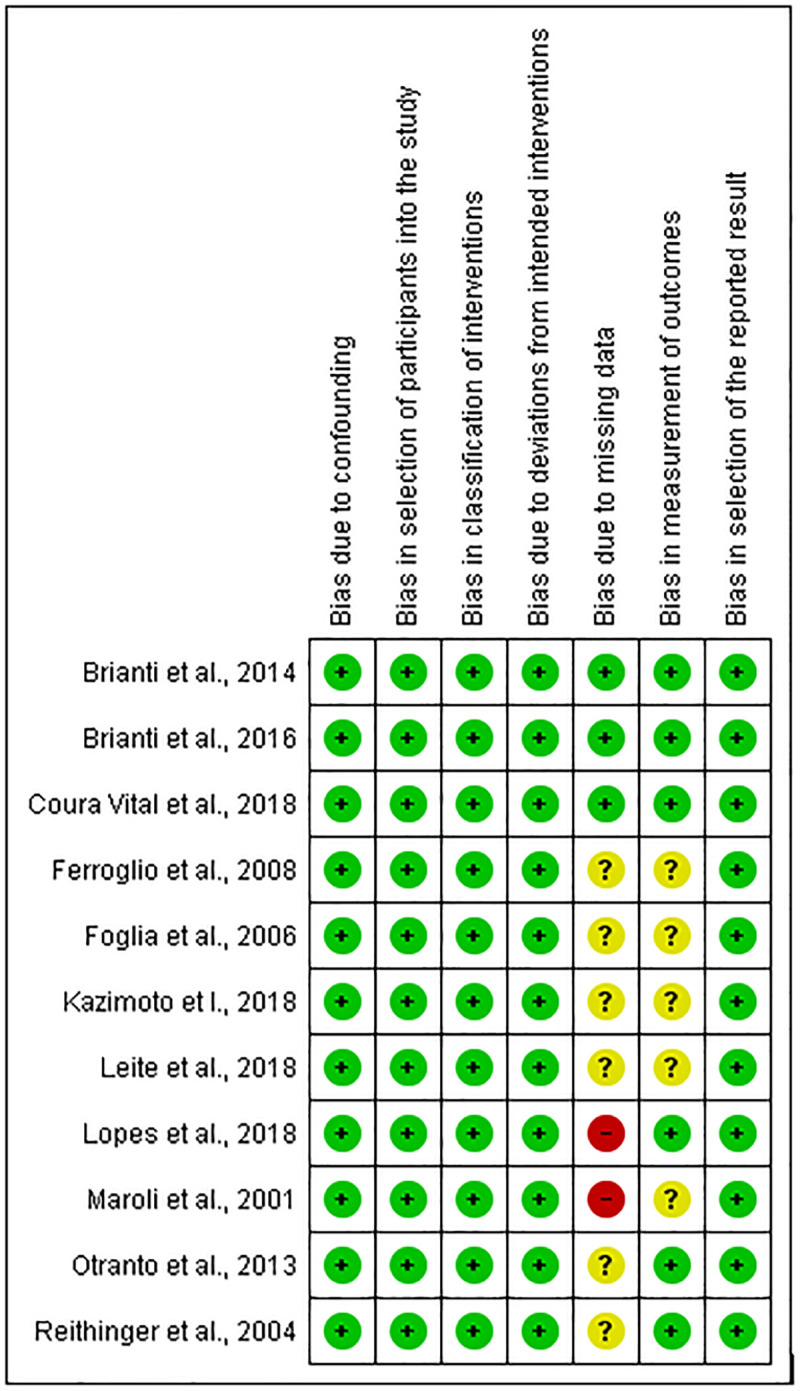
Summary of risk of bias assessment for non-randomized studies of interventions using ROB.1 tool. "+": low risk of bias; "?": unclear risk of bias; "−": high risk of bias.

A summary of 14 eligible studies (with 15 comparisons) [7, 14–16, 21–30] included in the current meta-analysis is provided in the S1 Table. Eleven studies assessed the effectiveness of DMC in reducing CanL incidence, two studies evaluated the effectiveness of Seresto, and one study assessed the effectiveness of both DMC and Seresto in controlling CanL. In total, 3786 collared dogs and 3427 uncollared dogs were included in the quantitative analysis. The follow-up duration of the included studies ranges from 5 months to 24 months, and the year of publication of the included studies is between 2001 and 2019. Due to differences in the claimed period of insecticidal activity of collars, DMC collars were replaced after 4–6 months of application [13], whereas Seresto collars were replaced after 6–8 months of application [31]. All of the studies included in this meta-analysis have been conducted in three countries, including Brazil, Iran, and Italy. Regarding the diagnostic methods, six studies used both serological and molecular methods while eight studies used one or two serological methods/s. The risk of bias summaries of included studies are showed in Figs 2 and 3. Of 14 studies, six, six, and two studies had an unclear risk of bias in the measurement of outcome, unclear risk of bias due to missing data, and high risk of bias due to missing data, respectively.

### Publication bias assessment

**Fig 4:**
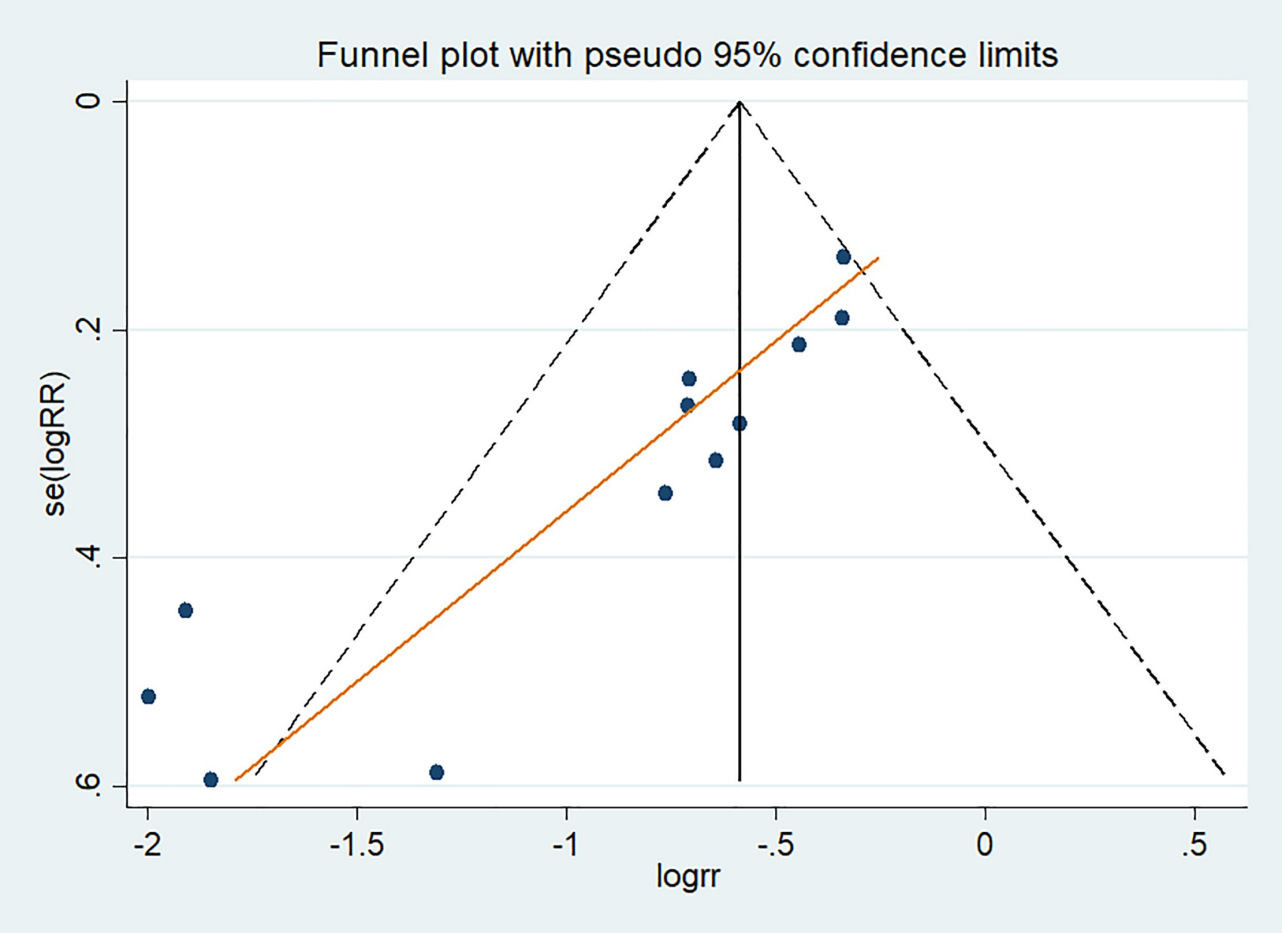
Funnel plot, using 12 studies that evaluated the effectiveness of deltamethrin-impregnated dog collars for controlling canine visceral leishmaniasis incidence, with log risk ratio(logRR) displayed on the horizontal axis, against its standard(se(logRR)) on the vertical axis.

In this study, the presence of publication bias among studies that used DMC for controlling CanL incidence was examined by visual inspection of the funnel plot and Egger's test for small-study effects. The funnel plot appears to be asymmetrical (Fig 4), and the presence of publication bias was statistically confirmed by Egger's test for small-study effects, with bias coefficient (B) = -3.356, 95%CI: -4.475 - -2.2378680 and *P* < 0.001. Since publication bias was detected, we performed Duval and Tweedie non-parametric/ trim and fill method [32]; however, the result of trim and fill adjusted RR (RR = 0.465, 95%CI, 0.358–0.604) was not different from the unadjusted pooled RR estimates (RR = 0.461, 95%CI, 0.353–0.604). So, we reported an unadjusted estimate of pooled RR. Because the number of included studies evaluating Seresto’s are small, it was impossible to evaluate publication bias.

### Sensitivity analysis

**Fig 5:**
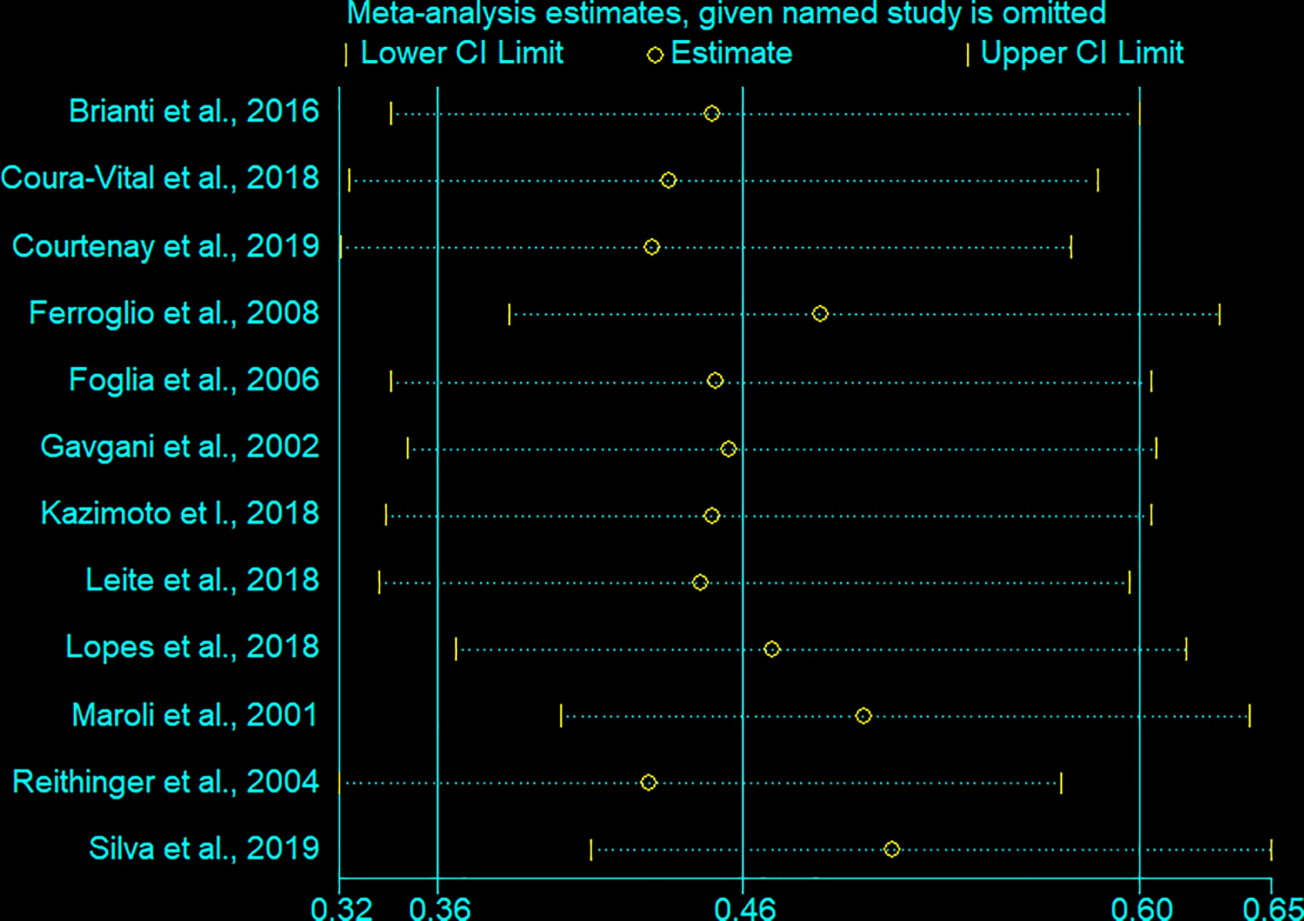
A leave-one-out sensitivity analysis forest plot of 12 studies that assessed the effectiveness of deltamethrin-impregnated dog collars in reducing canine visceral leishmaniasis incidence.

A leave-one-out sensitivity analysis was performed to determine the effect of a single study on the overall efficacy estimates between studies using DMC (Fig 5). A leave-one-out sensitivity analysis of the effectiveness of DMC in reducing CanL incidence ranges from 48.2% (when [30] removed) to 56.7% (when [29] removed), which is not considerably different from the overall efficacy of 54%. Due to an inadequate number of studies, we did not carry out a leave-one-out sensitivity analysis for Seresto's efficacy.

### Meta-analysis result

**Fig 6:**
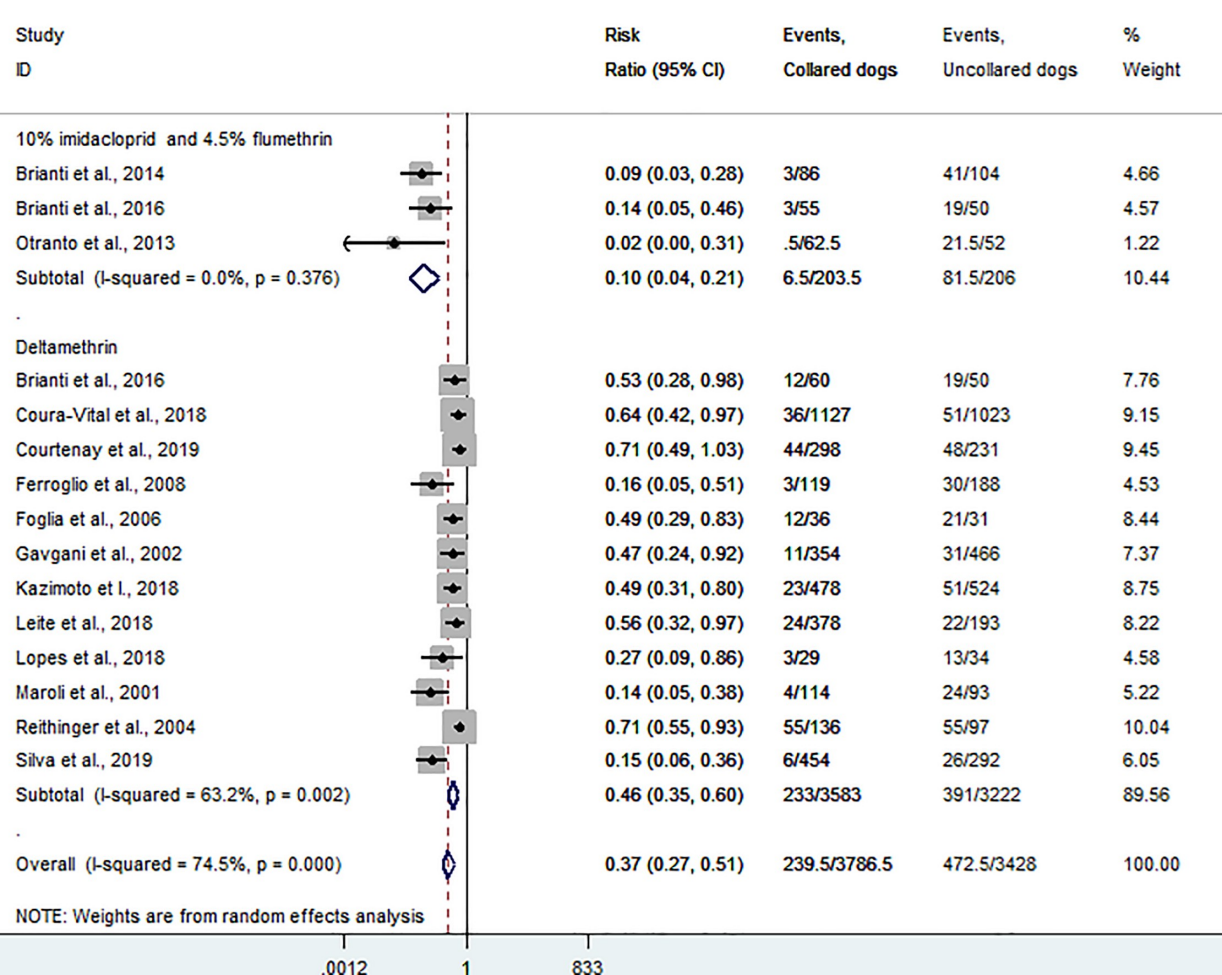
A forest plot showing effectiveness of insecticide-impregnated dog collars in reducing canine visceral leishmaniasis incidence.

The effectiveness of DMC and Seresto in reducing the risk of CanL was assessed in 12 and 3 studies, respectively. DMC use showed a 54% reduction in the incidence of CanL (RR = 0.461, 95% CI: 0.353–0.604), with statistically significant moderate heterogeneity among studies (I^2^ = 63.2%, *P* = 0.002). And according to combined results of 3 studies, the use of Seresto decreases CanL incidence by 90% in collared dogs relative to uncollared control dogs (RR = 0.098, 95%CI = 0.045–0.213, with no evidence of heterogeneity among studies and was statistically significant (*P* = 0.376, I^2^ = 0.0%) (Fig 6).

### Subgroup analysis

The result of the pooled effectiveness of DMC stratified by diagnostic methods and follow-up period are presented in Table 1. Subgroup analysis based on diagnostic methods revealed an overall RR = 0.303, 95%CI: 0.1–0.58) by one serological method, (RR = 0.450, 95%CI: 0.280–0.723) by two serological methods and (RR = 0.66, 95%CI: 0.53–0.83) by serological and molecular methods. According to subgroup analysis stratified by follow-up time, DMC decrease CanL incidence by 58% (RR = 0.42, 95%CI: 0.20–0.87), 54% (RR = 0.46, 95%CI: 0.31–0.68), 53% (RR = 0.47, 95%CI: 0.29–0.82) for follow-up periods of 5 to 6 months, 1 year and 2 years, respectively. Subgroup analysis was not performed for the effectiveness of Seresto in controlling the incidence of CanL due to similarities of diagnostic methods of included studies, and the country where the studies were conducted.

**Table 1:** Subgroup analysis stratified by diagnostic methods and follow-up period, from 2001 to 2019.

Characteristics	Number of studies	Collared dogs		Uncollared dogs		Pooled RR (95%CI)	I^2^ and P-value
		Number of positive	Total sample size	Number of positive	Total sample size		
Diagnostic methods							
one serological method	4	30	623	106	778	0.30(0.16–0.58)	63.2%& *P* = 0.002
two serological methods	4	89	2437	150	2032	0.45(0.28–0.72)	66.5%& *P* = 0.030
Serological and molecular methods	4	114	523	135	142	0.66(0.53–0.83)	10.1%& *P* = 0.343
Follow-up period							
5 to 6 months	3	84	1068	132	913	0.42(0.20–0.87)	63.2%&*P* = 0.002
1 year	5	65	1689	144	1761	0.46(0.31–0.68)	37.8%&*P* = 0.0169
2 years	4	84	826	111	548	0.47(0.29–0.78)	68%&*P* = 0.025

### Meta-regression analysis

Univariate meta-regression was conducted based on years of publication of included studies and a total sample size of 12 selected studies assessing the effectiveness of DMC on CanL incidence. However, both years of publication (meta-regression coefficient: .0031089, 95%CI: -.0468655 - .0530832), *P* = 0.893) and total sample size (meta-regression coefficient: .0001854; 95% CI: -.0004214 - .0007922); *P*  =  0.512) were found to be statistically non-significant predictors of effectiveness of DMC in reducing CanL incidence. Because of an insufficient number of studies, meta-regression analysis was impossible for Seresto efficacy.

## Discussion

While a previous systematic review (without meta-analysis) supports the use of insecticide-impregnated dog collars can prevent CanL [17], this study is, to the best of our knowledge, the first updated systematic review with a subsequent meta-analysis that synthesized evidence for the efficacy of insecticide-impregnated dog collars in reducing the incidence of CanL. In total, 14 studies (with 15 comparisons) with 3786 collared dogs and 3428 uncollared dogs were considered in this study. Pooled effectiveness results imply that both DMC and Seresto are substantially effective in decreasing the incidence of CanL by 54% and 90%, respectively compared with uncollared dogs. Thus, incorporation of insecticide-impregnated dog collars as a public health measure is a feasible alternative to complements existing CanL control activities, though further large-scale studies are needed to evaluate and monitor insecticide-impregnated dog collars effectiveness to achieve better success in decreasing incidence of CanL.

In the present systematic review and meta-analysis, pooled estimates of DMC effectiveness for reducing CanL incidence was 54%. This finding is in line with the findings of individual studies in Brazil, Italy, Iran that reported that DMC could reduce risk of CanL by 46–54% [14, 26, 27], 47–51% ([16, 25], and 53% [7], respectively. Also, a mathematical modeling study conducted in Brazil demonstrated that DMC was the most effective measure for CanL control, among other control measures [33]. The effectiveness of DMC for the reduction of CanL may be associated with its active ingredient dual properties of potent antifeeding effect and insecticidal effect. Studies have shown that DMC can reduce 85% contact between dogs and phlebotomine sand flies for 4–6 months, which in effect decreases infection in dogs and spreads to humans [1, 13]. DMC efficacy stratified by follow-up time was estimated to be 58% (RR = 0.42, 95%CI (0.20–0.87), 54% (RR = 0.46, 95%CI (0.31–0.68), 53% (RR = 0.47, 95%CI (0.29–0.82) for follow-up periods of 5 to 6 months, 1 year and 2 years, respectively. An increase in the follow-up period showed a slight decline in DMC efficacy which might be associated with collar loss. In Brazil, where most of the included studies have been conducted, the dog collar loss rate reaches up to 41% [29]. Consequently, dogs remain unprotected until subsequent dog collar replacement is performed.

Collar loss rate and collar replacement approaches would have led to the discrepancy in DMC and Seresto collars' effectiveness. The loss of collar rate in DMC studies was generally higher (34.5–56%) [23, 29, 34] than in the Seresto studies (losses were in general below 27%) [15]. Studies evaluating the efficacy of Seresto collars were conducted in controlled environments (kennels), where dogs were monitored daily for collar loss, and collars lost were replaced immediately. On the other hand, studies that investigated DMC effectiveness were conducted in community settings where dogs were not visited daily, and collar loss was replaced on 120 days, according to DMC collar brand recommendations. We could infer that low collar loss rate and better collar replacement strategies could have contributed to the higher effectiveness of Seresto collars in reducing the incidence of CanL.

In this systematic review and meta-analyses, meta-regression analysis did not show statistically significant association with both total sample size and year of publication, which can be interpreted as DMC efficacy in reducing the risk of incidental CanL was not influenced by total sample size newer studies.

The protective effect of Seresto against CanL is relatively less extensively studied and exclusively evaluated in Italy. Based on pooled estimates of this study, Seresto showed better effectiveness in the reduction of CanL incidence (90%), compared to DMC (54%). This comparatively better protection provided by Seresto could be due to the synergistic action of insecticidal properties of imidacloprid and the acaricidal properties of flumethrin, which ensure the slow and continuous release of active ingredients from collar matrix system to over skin surface of collared dogs, thereby provide 8 months of protection against the vector, although DMC conferred 4–6 months of protection against sandflies [31, 35]. Considerably greater protection observed in Seresto collar use than DMC collar may be influenced by the diagnostic methods implemented; for instance, all studies that assessed the effectiveness of Seresto collar used highly sensitive and specific molecular methods, whereas only four out of twelve studies that assessed DMC efficacy employed molecular methods. Another possible reason for the observed difference in protective effect could be related to variability in the disease transmission pattern between countries where included studies have been conducted. Seven of the twelve included studies that assessed DMC efficacy have been conducted in Brazil where CanL transmission shows variations across regions of its extensive territory. Also, no study that evaluated the effect of Seresto has been conducted in Brazil.

There are some limitations noted in this study. First, due to the relatively small number of included studies, assessment of publication bias and meta-regression analysis could be underpowered. Second, taking in to account the small studies included here to provide pooled estimates of Seresto effectiveness of for control of CanL incidence, there may be a lack of power in the overall effectiveness of Seresto. Therefore, the overall effectiveness of Seresto should be interpreted with caution. Third, while the loss of follow-up in a longitudinal study is inevitable, the included studies have reported high rate loss to follow-up which may induce attrition bias. Fourth, the studies included in this meta-analysis are from three countries. Fifth, this study did not include studies published other than the English language, which may lead to language bias. Despite these shortcomings, we performed an exhaustive and systematic literature search using databases and manual searches, we were able to use robust statistical techniques to combine overall effectiveness by enlarging sample size, we assessed sources of heterogeneity using both subgroup analysis and meta-regression, and we performed a sensitivity analysis to ensure that our estimate was not affected by a single study which could enable us to draw a firm conclusion.

## Conclusion

The result of the current evidence suggests that insecticide-impregnated dog collars could reduce the incidence rate of CanL. Consequently, insecticide-impregnated dog collars could be a valid alternative for inclusion in public health measures for reducing the incidence of CanL, which may in turn help to mitigate humans' zoonotic visceral leishmaniasis. Further large-scale studies that would evaluate and monitor the effectiveness of insecticide-impregnated dog collars are needed to provide a clearer picture of how much insecticide-impregnated dog collars may influence CanL incidence taking into account various socio-demographic and environmental factors.

## Supporting information

S1 Checklist PRISMA checklist. (DOC)

S1 Table Characteristics of selected studies. (DOCX)

S1 File Search strategy. (DOCX)

S2 File References of screened studies. (DOCX)
