# Supplementary material for: Effectiveness of insecticide-impregnated dog collars in reducing incidence rate of canine visceral leishmaniasis: A systematic review and meta-analysis
Source: PLoS One. 2020 Sep 3;15(9):e0238601. doi: 10.1371/journal.pone.0238601 (PMC7470253; doi:10.1371/journal.pone.0238601)
Supplement: S1 Table — (DOCX) [file pone.0238601.s002.docx]

**S1 Table** Characteristics of selected studies

| Study  S.N. | Authors | Study design | Country | Intervention | Length of follow up  (in months) | Diagnostic method | Intervention group (collared dogs) | | Control group  (uncollared dogs) | |
| --- | --- | --- | --- | --- | --- | --- | --- | --- | --- | --- |
|  |  |  |  |  |  |  | Number of positive | Total  Sample  size | Number of positive | Total  Sample  size |
| 1 | (Brianti et al., 2014) | Randomized interventional study | Italy | 10% imidacloprid and 4.5% flumethrin | 10 | IFAT, PCR & cytology | 3 | 86 | 41 | 104 |
| 2 | (Brianti et al., 2016) | Randomized interventional study | Italy | Deltamethrin | 12 | IFAT, PCR & cytology | 12 | 60 | 19 | 50 |
|  |  |  |  | 10% imidacloprid and 4.5% flumethrin | 12 | IFAT, PCR & cytology | 3 | 55 | 19 | 50 |
| 3 | (Coura-Vital et al., 2018) | Non-randomized interventional study | Brazil | Deltamethrin | 12 | DPP&  ELISA | 36 | 1127 | 51 | 1023 |
| 4 | (Courtenay et al., 2019) | Randomized interventional study | Brazil | Deltamethrin | 24 | ELISA& PCR | 44 | 298 | 48 | 231 |
| 5 | (Ferroglio, Poggi, & Trisciuoglio, 2008) | Non-randomized interventional study | Italy | Deltamethrin | 12 | IFAT | 3 | 119 | 30 | 188 |
| 6 | (Foglia Manzillo et al., 2006) | Non-randomized interventional study | Italy | Deltamethrin | 24 | IFAT | 12 | 36 | 21 | 31 |
| 7 | (Gavgani et al., 2002) | Randomized interventional study | Iran | Deltamethrin | 12 | DAT | 11 | 354 | 31 | 466 |
| 8 | (Kazimoto et al., 2018) | Randomized interventional study | Brazil | Deltamethrin | 6 | DPP& EIE | 23 | 478 | 51 | 524 |
| 9 | (Leite et al., 2018) | Non-randomized interventional study | Brazil | Deltamethrin | 24 | DPP& EIE | 24 | 378 | 22 | 193 |
| 10 | (Lopes et al., 2018) | Non-randomized interventional study | Brazil | Deltamethrin | 12 | DPP, ELISA & PCR | 3 | 29 | 13 | 34 |
| 11 | (Maroli, Mizzoni, Siragusa, D'Orazi, & Gradoni, 2001) | Non-randomized interventional study | Italy | Deltamethrin | 24 | IFAT&parasitology | 4 | 114 | 24 | 93 |
| 12 | (Otranto et al., 2013) | Non-randomized interventional study | Italy | 10% imidacloprid and 4.5% flumethrin | 24 | IFAT, PCR & cytology | 0 | 62 | 21 | 51 |
| 13 | (Reithinger et al., 2004) | Non-randomized interventional study | Brazil | Deltamethrin | 5 | ELISA& PCR | 55 | 136 | 55 | 97 |
| 14 | (Silva et al., 2019) | Randomized interventional study | Brazil | Deltamethrin | 6 | DPP&EIE | 6 | 454 | 26 | 292 |

**Aberrations:** indirect immunofluorescent antibody test (IFAT); Dual Path Platform (DPP); Enzyme-Linked Immunosorbent Assay (ELISA); direct agglutination test (DAT); EIE-leishmaniose-visceral-canina-Bio-Manguinhos (EIE-LVC)
