# Supplementary material for: Effectiveness of insecticide-impregnated dog collars in reducing incidence rate of canine visceral leishmaniasis: A systematic review and meta-analysis
Source: PLoS One. 2020 Sep 3;15(9):e0238601. doi: 10.1371/journal.pone.0238601 (PMC7470253; doi:10.1371/journal.pone.0238601)
Supplement: S1 File — (DOCX) [file pone.0238601.s003.docx]

Search strategies: details of search strategy.

Scopus (search result =41)

( ( TITLE-ABS ( canine AND leishmaniasis ) ) OR ( TITLE-ABS ( canine AND leishmaniosis ) ) OR ( TITLE-ABS ( canine AND visceral AND leishmaniasis ) ) OR ( TITLE-ABS ( zoonotic AND visceral AND leishmaniasis ) ) ) AND ( TITLE-ABS ( "dog collars" OR "4% deltamethrin" OR "insecticide-impregnated dog collars" OR "deltamethrin-impregnated dog collars" OR "deltamethrin" OR "10% imidacloprid and 4.5% flumethrin" OR "imidacloprid and flumethrin" OR "Flumethrin-Imidacloprid Collar" ) )

PubMed(search result =27)

Search (("canine leishmaniasis"[Title/Abstract] OR "canine leishmaniosis "[Title/Abstract] OR "canine visceral leishmaniasis"[Title/Abstract] OR "zoonotic visceral leishmaniasis"[Title/Abstract])) AND ("dog collars"[Title/Abstract] OR "4% deltamethrin"[Title/Abstract] OR "insecticide-impregnated dog collars"[Title/Abstract] OR "deltamethrin-impregnated dog collars"[Title/Abstract] OR "deltamethrin"[Title/Abstract] OR "10% imidacloprid[Title/Abstract] AND 4.5% flumethrin"[Title/Abstract] OR "imidacloprid[Title/Abstract] AND flumethrin"[Title/Abstract] OR "Flumethrin-Imidacloprid Collar"[Title/Abstract])

Embase(search result =2)

No. Query Results Results

#15. #5 AND #14 62

#14. #6 OR #7 OR #8 OR #9 OR #10 OR #11 OR #12 OR #13 4,825

#13. 'flumethrin imidacloprid' AND collar 2

#12. imidacloprid AND flumethrin 49

#11. 10% AND imidacloprid AND 4.5% AND flumethrin 27

#10. 'deltamethrin' 4,594

#9. 'deltamethrin impregnated' AND dog AND collars 23

#8. 'insecticide impregnated' AND dog AND collars 19

#7. 4% AND deltamethrin 3,996

#6. dog AND collar 224

#5. #1 OR #2 OR #3 OR #4 2,576

#4. zoonotic AND visceral AND leishmaniasis 563

#3. 'canine visceral leishmaniasis' 660

#2. 'canine leishmaniosis' 325

#1. ('canine'/exp OR canine) AND ('leishmaniasis'/exp 2,223

OR leishmaniasis)

Web of Science (search result =80)

# 1(results=2,399)

TS=("canine leishmaniasis" OR "canine leishmaniosis " OR "canine visceral leishmaniasis" OR "zoonotic visceral leishmaniasis")

Indexes=SCI-EXPANDED, SSCI, A&HCI, CPCI-S, CPCI-SSH, BKCI-S, BKCI-SSH, ESCI, CCR-EXPANDED, IC Timespan=All years

# 2(results=5,083)

TS=( "dog collars" OR "4% deltamethrin" OR "insecticide-impregnated dog collars" OR "deltamethrin-impregnated dog collars" OR "deltamethrin" OR "10% imidacloprid and 4.5% flumethrin" OR "imidacloprid and flumethrin" OR "Flumethrin-Imidacloprid Collar" )

Indexes=SCI-EXPANDED, SSCI, A&HCI, CPCI-S, CPCI-SSH, BKCI-S, BKCI-SSH, ESCI, CCR-EXPANDED, IC Timespan=All years

#2 AND #1(results=80)

Indexes=SCI-EXPANDED, SSCI, A&HCI, CPCI-S, CPCI-SSH, BKCI-S, BKCI-SSH, ESCI, CCR-EXPANDED, IC Timespan=All years

Ovid Medline(R) ( search results =29)

1(results =1522)

("canine leishmaniasis" or "canine leishmaniosis " or "canine visceral leishmaniasis" or "zoonotic visceral leishmaniasis").mp. [mp=title, abstract, original title, name of substance word, subject heading word, floating sub-heading word, keyword heading word, organism supplementary concept word, protocol supplementary concept word, rare disease supplementary concept word, unique identifier, synonyms]

2(results =3068 )

("dog collars" or "4% deltamethrin" or "insecticide-impregnated dog collars" or "deltamethrin-impregnated dog collars" or "deltamethrin" or "10% imidacloprid and 4.5% flumethrin" or "imidacloprid and flumethrin" or "Flumethrin-Imidacloprid Collar").mp. [mp=title, abstract, original title, name of substance word, subject heading word, floating sub-heading word, keyword heading word, organism supplementary concept word, protocol supplementary concept word, rare disease supplementary concept word, unique identifier, synonyms]

1 and 2(results =29)
