# Supplementary material for: Effectiveness of insecticide-impregnated dog collars in reducing incidence rate of canine visceral leishmaniasis: A systematic review and meta-analysis
Source: PLoS One. 2020 Sep 3;15(9):e0238601. doi: 10.1371/journal.pone.0238601 (PMC7470253; doi:10.1371/journal.pone.0238601)
Supplement: S2 File — (DOCX) [file pone.0238601.s004.docx]

References of screened studies

1. Alexander B, Maroli M. Control of phlebotomine sandflies. Medical and Veterinary Entomology. 2003;17(1):1-18. doi: 10.1046/j.1365-2915.2003.00420.x. PubMed PMID: WOS:000182117700001.

2. Alvar J, Cañavate C, Molina R, Moreno J, Nieto J. Canine leishmaniasis. 2004. p. 1-88.

3. Amora SSA, Bevilaqua CML, Feijo FMC, Alves ND, Maciel MD. Control of Phlebotomine (Diptera: Psychodidae) Leishmaniasis Vectors. Neotropical Entomology. 2009;38(3):303-10. doi: 10.1590/s1519-566x2009000300001. PubMed PMID: WOS:000268141500001.

4. Aoun K, Chouihi E, Boufaden I, Mahmoud R, Bouratbine A, Bedoui K. Efficacy of Deltamethrine-impregnated collars Scalibor in the prevention of canine leishmaniasis in the area of Tunis. Archives de l'Institut Pasteur de Tunis. 2008;85(1-4):63-8.

5. Barbosa MAG, Alexandre-Pires G, Soares-Clemente M, Marques C, Rodrigues OR, De Brito TV, et al. Cytokine Gene Expression in the Tissues of Dogs Infected by Leishmania infantum. Journal of Comparative Pathology. 2011;145(4):336-44. doi: 10.1016/j.jcpa.2011.03.001.

6. Batista LFS, Utsunomiya YT, Silva TBF, Carneiro MM, Paiva JSF, Silva RB, et al. Canine leishmaniasis: Genome-wide analysis and antibody response to Lutzomyia longipalpis saliva. PLoS ONE. 2018;13(5). doi: 10.1371/journal.pone.0197215.

7. Berg C, Botner A, Browman H, De Koeijer A, Depner K, Domingo M, et al. Scientific Opinion on canine leishmaniosis. Efsa Journal. 2015;13(4). doi: 10.2903/j.efsa.2015.4075. PubMed PMID: WOS:000409028000013.

8. Boggiatto PM, Gibson-Corley KN, Metz K, Gallup JM, Hostetter JM, Mullin K, et al. Transplacental Transmission of Leishmania infantum as a Means for Continued Disease Incidence in North America. Plos Neglected Tropical Diseases. 2011;5(4). doi: 10.1371/journal.pntd.0001019. PubMed PMID: WOS:000289937400016.

9. Bourdeau P, Saridomichelakis MN, Oliveira A, Oliva G, Kotnik T, Galvez R, et al. Management of canine leishmaniosis in endemic SW European regions: a questionnaire-based multinational survey. Parasites & Vectors. 2014;7. doi: 10.1186/1756-3305-7-110. PubMed PMID: WOS:000335341700001.

10. Brianti E, Gaglio G, Napoli E, Falsone L, Prudente C, Basano FS, et al. Efficacy of a slow-release imidacloprid (10%)/flumethrin (4.5%) collar for the prevention of canine leishmaniosis. Parasites & Vectors. 2014;7. doi: 10.1186/1756-3305-7-327. PubMed PMID: WOS:000339288200001.

11. Brianti E, Napoli E, Gaglio G, Falsone L, Giannetto S, Basano FS, et al. Field Evaluation of Two Different Treatment Approaches and Their Ability to Control Fleas and Prevent Canine Leishmaniosis in a Highly Endemic Area. Plos Neglected Tropical Diseases. 2016;10(9). doi: 10.1371/journal.pntd.0004987. PubMed PMID: WOS:000385627900042.

12. Carvalho AG, Luz JGG, Rodrigues LD, Dias JVL, Fontes CJF. Knowledge and preventive attitudes on visceral leishmaniasis among Brazilian dog owners: Report from a household survey. Transactions of the Royal Society of Tropical Medicine and Hygiene. 2019;113:S103. doi: 10.1093/trstmh/trz090.

13. Cassini R, Signorini M, di Regalbono AF, Natale A, Montarsi F, Zanaica M, et al. Preliminary study of the effects of preventive measures on the prevalence of Canine Leishmaniosis in a recently established focus in northern Italy. 2013. p. 157-61.

14. Chaskopoulou A, Miaoulis M, Kashefi J. Ground ultra low volume (ULV) space spray applications for the control of wild sand fly populations (Psychodidae: Phlebotominae) in Europe. Acta Tropica. 2018;182:54-9. doi: 10.1016/j.actatropica.2018.02.003.

15. Costa D, Codeco CT, Bermudi PMM, Rodas LAC, Nunes CM, Hiramoto RM, et al. Control of canine visceral leishmaniasis by euthanasia: estimated effect based on a survey and mathematical modeling. Cadernos De Saude Publica. 2020;36(2). doi: 10.1590/0102-311x00221418. PubMed PMID: WOS:000518811200001.

16. Coura FM, de Oliveira Paes Leme F, dos Santos Alves F, de Araujo RB, da Costa Val Bicalho AP. Evaluation of the antifeeding and insecticidal effects of a deltamethrin-impregnated collar on Lutzomyia longipalpis. Acta Veterinaria Brasilica. 2019;13(4):192-7. doi: 10.21708/avb.2019.13.4.8331.

17. Coura-Vital W, Leal GGD, Marques LA, Pinheiro AD, Carneiro M, Reis AB. Effectiveness of deltamethrin-impregnated dog collars on the incidence of canine infection by Leishmania infantum: A large scale intervention study in an endemic area in Brazil. Plos One. 2018;13(12). doi: 10.1371/journal.pone.0208613. PubMed PMID: WOS:000452644700037.

18. Courtenay O, Bazmani A, Parvizi P, Ready PD, Cameron MM. Insecticide–impregnated dog collars reduce infantile clinical visceral leishmaniasis under operational conditions in NW Iran: A community–wide cluster randomised trial. PLoS Neglected Tropical Diseases. 2019;13(3). doi: 10.1371/journal.pntd.0007193.

19. Courtenay O, Dilger E, Calvo-Bado LA, Kravar-Garde L, Carter V, Bell MJ, et al. Sand fly synthetic sex-aggregation pheromone co-located with insecticide reduces the incidence of infection in the canine reservoir of visceral leishmaniasis: A stratified cluster randomised trial. PLoS Neglected Tropical Diseases. 2019;13(10). doi: 10.1371/journal.pntd.0007767.

20. Courtenay O, Gavgani SM, Bazmani A, Cameron M, Davies CR. Insecticide-impregnated dog collars reduce human visceral leishmaniasis under operational conditions in NW Iran: A community-wide cluster randomised trial. Tropical Medicine and International Health. 2017;22:349. doi: 10.1111/(ISSN)1365-3156.

21. Courtenay O, Gillingwater K, Gomes PAF, Garcez LM, Davies CR. Deltamethrin-impregnated bednets reduce human landing rates of sandfly vector Lutzomyia longipalpis in Amazon households. Medical and Veterinary Entomology. 2007;21(2):168-76. doi: 10.1111/j.1365-2915.2007.00678.x. PubMed PMID: WOS:000247175100006.

22. Courtenay O, Kovacic V, Gomes PAF, Garcez LM, Quinnell RJ. A long-lasting topical deltamethrin treatment to protect dogs against visceral leishmaniasis. Medical and Veterinary Entomology. 2009;23(3):245-56. doi: 10.1111/j.1365-2915.2009.00815.x. PubMed PMID: WOS:000268970800009.

23. Cutolo AA, Galvis-Ovallos F, Neves ED, Silva FO, Chester ST, Fankhauser B. Repellent efficacy of a new combination of fipronil and permethrin against Lutzomyia longipalpis. Parasites & Vectors. 2018;11. doi: 10.1186/s13071-018-2831-7. PubMed PMID: WOS:000430810400004.

24. Dantas-Torres F. Canine leishmaniosis in South America. Parasites & Vectors. 2009;2. doi: 10.1186/1756-3305-2-s1-s1. PubMed PMID: WOS:000270371800001.

25. Dantas-Torres F, Nogueira FD, Menz I, Tabanez P, da Silva SM, Ribeiro VM, et al. Vaccination against canine leishmaniasis in Brazil. International Journal for Parasitology. 2020;50(3):171-6. doi: 10.1016/j.ijpara.2020.01.001. PubMed PMID: WOS:000527355500001.

26. David JR, Stamm LM, Bezerra HS, Souza RN, Killick-Kendrick R, Lima JWO. Deltamethrin-impregnated dog collars have a potent anti-feeding and insecticidal effect on Lutzomyia longipalpis and Lutzomyia migonei. Memorias Do Instituto Oswaldo Cruz. 2001;96(6):839-47. doi: 10.1590/s0074-02762001000600018. PubMed PMID: WOS:000170361700018.

27. Davoust B, Roqueplo C, Parzy D, Watier-Grillot S, Marie J-L. A twenty-year follow-up of canine leishmaniosis in three military kennels in southeastern France. Parasites & vectors. 2013;6(1):323. doi: https://dx.doi.org/10.1186/1756-3305-6-323.

28. De Oliveira RD, Ribas G, Moura ACJ, Da Costa Val AP. Permethrin and deltamethrin: Comparison of efficiency in control of causing sandflies leishmaniasis canine visceral. Acta Veterinaria Brasilica. 2013;7(SUPPL. 1):586-8.

29. Dedet JP. Leishmaniasis: update. Presse médicale (Paris, France : 1983). 2000;29(18):1019-26.

30. Dereure J, Vanwambeke SO, Malé P, Martinez S, Pratlong F, Balard Y, et al. The potential effects of global warming on changes in canine leishmaniasis in a focus outside the classical area of the disease in Southern France. Vector-Borne and Zoonotic Diseases. 2009;9(6):687-94. doi: 10.1089/vbz.2008.0126.

31. Dilger E, Carter V, Gonzalez De Heredia M, Borges-Alves G, Nunes C, Garcez LM, et al. Manipulation of sandfly distribution within the peridomestic environment, and implications for the control of vectorborne disease. Tropical Medicine and International Health. 2017;22:165-6. doi: 10.1111/(ISSN)1365-3156.

32. Farkas R, Tanczos B. Canine leishmaniosis and its importance in Europe. Literature review. Magyar Allatorvosok Lapja. 2009;131(5):304-12. PubMed PMID: WOS:000266456800008.

33. Faucher B, Piarroux R. Visceral leishmaniasis: An update. Revue De Medecine Interne. 2011;32(9):544-51. doi: 10.1016/j.revmed.2010.08.002. PubMed PMID: WOS:000294835100004.

34. Fernandez M, Tabar MD, Arcas A, Mateu C, Homedes J, Roura X. Comparison of efficacy and safety of preventive measures used against canine leishmaniasis in southern European countries: Longitudinal retrospective study in 1647 client-owned dogs (2012-2016). Veterinary Parasitology. 2018;263:10-7. doi: 10.1016/j.vetpar.2018.09.014. PubMed PMID: WOS:000450382100003.

35. Ferroglio E, Poggi M, Trisciuoglio A. Evaluation of 65% permethrin spot-on and deltamethrin-impregnated collars for canine Leishmania infantum infection prevention. Zoonoses and Public Health. 2008;55(3):145-8. doi: 10.1111/j.1863-2378.2007.01092.x. PubMed PMID: WOS:000253826900004.

36. Fèvre EM, Bronsvoort BMDC, Hamilton KA, Cleaveland S. Animal movements and the spread of infectious diseases. Trends in Microbiology. 2006;14(3):125-31. doi: 10.1016/j.tim.2006.01.004.

37. Foglia Manzillo V, Oliva G, Pagano A, Manna L, Maroli M, Gradoni L. Deltamethrin-impregnated collars for the control of canine leishmaniasis: Evaluation of the protective effect and influence on the clinical outcome of Leishmania infection in kennelled stray dogs. Veterinary Parasitology. 2006;142(1-2):142-5. doi: 10.1016/j.vetpar.2006.06.029.

38. Galvez R, Montoya A, Fontal F, De Murguia LM, Miro G. Controlling phlebotomine sand flies to prevent canine Leishmania infantum infection: A case of knowing your enemy. Research in Veterinary Science. 2018;121:94-103. doi: 10.1016/j.rvsc.2018.10.008. PubMed PMID: WOS:000451937600014.

39. Gavgani ASM, Hodjati MH, Mohite H, Davies CR. Effect of insecticide-impregnated dog collars on incidence of zoonotic visceral leishmaniasis in Iranian children: a matched-cluster randomised trial. Lancet. 2002;360(9330):374-9. doi: 10.1016/s0140-6736(02)09609-5. PubMed PMID: WOS:000177255600011.

40. Genta FA, Diaz-Albiter HM, Salgueiro P, Gomes B. Control of Vector-Borne Human Parasitic Diseases. Biomed Research International. 2016. doi: 10.1155/2016/1014805. PubMed PMID: WOS:000391592900001.

41. Gomez SA, Chapman LAC, Dilger E, Courtenay O, Picado A. Estimating the efficacy of community-wide use of systemic insecticides in dogs to control zoonotic visceral leishmaniasis: A modelling study in a Brazilian scenario. Plos Neglected Tropical Diseases. 2018;12(9). doi: 10.1371/journal.pntd.0006797. PubMed PMID: WOS:000446054600047.

42. Goyena E, Perez-Cutillas P, Chitimia L, Risueno J, Garcia-Martinez JD, Bernal LJ, et al. A cross-sectional study of the impact of regular use of insecticides in dogs on Canine Leishmaniosis seroprevalence in southeast Spain. Preventive Veterinary Medicine. 2016;124:78-84. doi: 10.1016/j.prevetmed.2015.12.009. PubMed PMID: WOS:000370457900010.

43. Gramiccia M. Recent advances in leishmaniosis in pet animals: Epidemiology, diagnostics and anti-vectorial prophylaxis. Veterinary Parasitology. 2011;181(1):23-30. doi: 10.1016/j.vetpar.2011.04.019.

44. Gramiccia M, Gradoni L. The current status of zoonotic leishmaniases and approaches to disease control. International Journal for Parasitology. 2005;35(11-12):1169-80. doi: 10.1016/j.ijpara.2005.07.001. PubMed PMID: WOS:000232901200003.

45. Gramiccia M, Gradoni L. The leishmaniases of Southern Europe. In: Takken W, Knols BGJ, editors. Emerging Pests and Vector-Borne Diseases in Europe. Ecology and Control of Vector-Borne Diseases. 12007. p. 75-95.

46. Grimaldi G, Teva A, dos-Santos CB, Santos FN, Pinto ID, Fux B, et al. Field trial of efficacy of the Leish-tec((R)) vaccine against canine leishmaniasis caused by Leishmania infantum in an endemic area with high transmission rates. Plos One. 2017;12(9). doi: 10.1371/journal.pone.0185438. PubMed PMID: WOS:000411980300068.

47. Guan L. Current status of kala-azar and vector control in China. Bulletin of the World Health Organization. 1991;69(5):595-601.

48. Halbig P, Hodjati MH, Mazloumi-Gavgani AS, Mohite H, Davies CR. Further evidence that deltamethrin-impregnated collars protect domestic dogs from sandfly bites. Medical and Veterinary Entomology. 2000;14(2):223-6. doi: 10.1046/j.1365-2915.2000.00229.x. PubMed PMID: WOS:000087562500015.

49. Ikeda-Garcia FA, Lopes RS, Marques FJ, de Lima VMF, Morinishi CK, Bonello FL, et al. Clinical and parasitological evaluation of dogs naturally infected by Leishmania (Leishmania) chagasi submitted to treatment with meglumine antimoniate. Veterinary Parasitology. 2007;143(3-4):254-9. doi: 10.1016/j.vetpar.2006.08.019. PubMed PMID: WOS:000244440500008.

50. Jacobson RL. Leishmaniasis in an era of conflict in the Middle East. Vector-Borne and Zoonotic Diseases. 2011;11(3):247-58. doi: 10.1089/vbz.2010.0068.

51. Kazimoto TA, Amora SSA, Figueiredo FB, Magalhaes JME, Freitas YBN, Sousa MLR, et al. Impact of 4% Deltamethrin-Impregnated Dog Collars on the Prevalence and Incidence of Canine Visceral Leishmaniasis. Vector borne and zoonotic diseases (Larchmont, NY). 2018;18(7):356-63. doi: https://dx.doi.org/10.1089/vbz.2017.2166.

52. Killick-Kendrick R, Killick-Kendrick M, Focheux C, Dereure J, Puech MP, Cadiergues MC. Protection of dogs from bites of phlebotomine sandflies by deltamethrin collars for control of canine leishmaniasis. Medical and Veterinary Entomology. 1997;11(2):105-11. doi: 10.1111/j.1365-2915.1997.tb00298.x.

53. Leite BMM, Solca MDS, Santos LCS, Coelho LB, Amorim L, Donato LE, et al. The mass use of deltamethrin collars to control and prevent canine visceral leishmaniasis: A field effectiveness study in a highly endemic area. PLoS neglected tropical diseases. 2018;12(5):e0006496. Epub 2018/05/15. doi: 10.1371/journal.pntd.0006496. PubMed PMID: 29758031; PubMed Central PMCID: PMCPMC5993122.

54. Lopes EG, Sevá AP, Ferreira F, Nunes CM, Keid LB, Hiramoto RM, et al. Vaccine effectiveness and use of collar impregnated with insecticide for reducing incidence of Leishmania infection in dogs in an endemic region for visceral leishmaniasis, in Brazil. Epidemiology and Infection. 2018;146(3):401-6. doi: 10.1017/S0950268817003053.

55. Maia C, Cardoso L. Spread of Leishmania infantum in Europe with dog travelling. Veterinary Parasitology. 2015;213(1-2):2-11. doi: 10.1016/j.vetpar.2015.05.003.

56. Marcondes M, Day MJ. Current status and management of canine leishmaniasis in Latin America. Research in Veterinary Science. 2019;123:261-72. doi: 10.1016/j.rvsc.2019.01.022. PubMed PMID: WOS:000463125600042.

57. Maroli M, Gradoni L, Oliva G, Castagnaro M, Crotti A, Lubas G, et al. Canine Leishmaniasis: guidelines for diagnosis, staging, therapy, monitoring and prevention. Part III: Prevention. Veterinaria. 2009;23(4):19-26. PubMed PMID: WOS:000269959200004.

58. Maroli M, Gradoni L, Oliva G, Castagnaro M, Crotti A, Lubas G, et al. Guidelines for prevention of leishmaniasis in dogs. Javma-Journal of the American Veterinary Medical Association. 2010;236(11):1200-6. doi: 10.2460/javma.236.11.1200. PubMed PMID: WOS:000277983800027.

59. Maroli M, Khoury C. Prevention and control of leishmaniasis vectors: Current approaches. Parassitologia. 2004;46(1-2):211-5.

60. Maroli M, Mizzon V, Siragusa C, D'Oorazi A, Gradoni L. Evidence for an impact on the incidence of canine leishmaniasis by the mass use of deltamethrin-impregnated dog collars in southern Italy. Medical and veterinary entomology. 2001;15(4):358-63. Epub 2002/01/05. doi: 10.1046/j.0269-283x.2001.00321.x. PubMed PMID: 11776454.

61. Miro G, Cardoso L, Pennisi MG, Oliva G, Baneth G. Canine leishmaniosis - new concepts and insights on an expanding zoonosis: part two. Trends in Parasitology. 2008;24(8):371-7. doi: 10.1016/j.pt.2008.05.003. PubMed PMID: WOS:000258522300009.

62. Miro G, Lopez-Velez R. Clinical management of canine leishmaniosis versus human leishmaniasis due to Leishmania infantum: Putting "One Health" principles into practice. Veterinary Parasitology. 2018;254:151-9. doi: 10.1016/j.vetpar.2018.03.002. PubMed PMID: WOS:000431160900025.

63. Miro G, Petersen C, Cardoso L, Bourdeau P, Baneth G, Solano-Gallego L, et al. Novel Areas for Prevention and Control of Canine Leishmaniosis. Trends in Parasitology. 2017;33(9):718-30. doi: 10.1016/j.pt.2017.05.005. PubMed PMID: WOS:000410017200009.

64. Molina R, Espinosa-Gongora C, Galvez R, Montoya A, Descalzo MA, Jimenez MI, et al. Efficacy of 65% permethrin applied to dogs as a spot-on against Phlebotomus perniciosus. Veterinary Parasitology. 2012;187(3-4):529-33. doi: 10.1016/j.vetpar.2012.01.024. PubMed PMID: WOS:000307323700025.

65. Moritz A, Prinzinger S, Bauer N. Canine visceral leishmaniasis: Infectious agent, infection, clinical signs, diagnosis, therapy and prophylaxis - a review. Kleintierpraxis. 2001;46(9):533-+. PubMed PMID: WOS:000171153600001.

66. Mueller RS, Rosenkrantz W, Bensignor E, Karaś-Tęcza J, Paterson T, Shipstone MA. Diagnosis and treatment of demodicosis in dogs and cats: Clinical consensus guidelines of the World Association for Veterinary Dermatology. Veterinary Dermatology. 2020;31(1):5-27. doi: 10.1111/vde.12806.

67. Noli C, Auxilia ST. Treatment of canine Old World visceral leishmaniasis: a systematic review. Veterinary Dermatology. 2005;16(4):213-32. doi: 10.1111/j.1365-3164.2005.00460.x. PubMed PMID: WOS:000231221900001.

68. Otranto D, Dantas-Torres F. The prevention of canine leishmaniasis and its impact on public health. Trends in Parasitology. 2013;29(7):339-45. doi: 10.1016/j.pt.2013.05.003. PubMed PMID: WOS:000321883300006.

69. Otranto D, Dantas-Torres F, de Caprariis D, Di Paola G, Tarallo VD, Latrofa MS, et al. Prevention of Canine Leishmaniosis in a Hyper-Endemic Area Using a Combination of 10% Imidacloprid/4.5% Flumethrin. Plos One. 2013;8(2). doi: 10.1371/journal.pone.0056374. PubMed PMID: WOS:000316849500013.

70. Palatnik-de-Sousa CB, Day MJ. One Health: The global challenge of epidemic and endemic leishmaniasis. Parasites & Vectors. 2011;4. doi: 10.1186/1756-3305-4-197. PubMed PMID: WOS:000296919000001.

71. Papadopoulos E, Angelou A, Diakou A, Halos L, Beugnet F. Five-month serological monitoring to assess the effectiveness of permethrin/fipronil (Frontline Tri-Act(R)) spot-on in reducing the transmission of Leishmania infantum in dogs. Veterinary parasitology, regional studies and reports. 2017;7:48-53. Epub 2017/01/01. doi: 10.1016/j.vprsr.2016.12.005. PubMed PMID: 31014657.

72. Papadopoulos E, Angelou A, Diakou A, Halos L, Beugnet F. Five-month serological monitoring to assess the effectiveness of permethrin/fipronil (Frontline Tri-Act®) spot-on in reducing the transmission of Leishmania infantum in dogs. Veterinary Parasitology: Regional Studies and Reports. 2017;7:48-53. doi: 10.1016/j.vprsr.2016.12.005.

73. Parin U, Erbas G, Ural K, Savasan S, Yuksel HT, Balat G, et al. Investigation of bacterial and fungal agents from cutaneous lesions in canine Leishmaniasis. Indian Journal of Animal Research. 2020;54(1):96-100. doi: 10.18805/ijar.B-696.

74. Passantino A, Russo M, Coluccio P. Canine leishmaniosis and euthanasia in Italy: a critical legal-ethical analysis. Revue Scientifique Et Technique-Office International Des Epizooties. 2010;29(3):537-48. PubMed PMID: WOS:000287993600009.

75. Paulin S, Frenais R, Thomas E, Baldwin PM. Laboratory assessment of the anti-feeding effect for up to 12 months of a slow release deltamethrin collar (Scalibor (R)) against the sand fly Phlebotomus perniciosus in dogs. Parasites & Vectors. 2018;11. doi: 10.1186/s13071-018-3094-z. PubMed PMID: WOS:000445964400002.

76. Petersen CA. Leishmaniasis, an Emerging Disease Found in Companion Animals in the United States. Topics in Companion Animal Medicine. 2009;24(4):182-8. doi: 10.1053/j.tcam.2009.06.006. PubMed PMID: WOS:000279654700004.

77. Petersen CA, Barr SC. Canine Leishmaniasis in North America: Emerging or Newly Recognized? Veterinary Clinics of North America-Small Animal Practice. 2009;39(6):1065-+. doi: 10.1016/j.cvsm.2009.06.008. PubMed PMID: WOS:000276005900006.

78. Quinnell RJ, Courtenay O. Transmission, reservoir hosts and control of zoonotic visceral leishmaniasis. Parasitology. 2009;136(14):1915-34. doi: 10.1017/s0031182009991156. PubMed PMID: WOS:000273515200006.

79. Ready PD. Leishmaniasis emergence and climate change. Revue Scientifique Et Technique-Office International Des Epizooties. 2008;27(2):399-412. doi: 10.20506/rst.27.2.1803. PubMed PMID: WOS:000259353700010.

80. Reguera RM, Morán M, Pérez-Pertejo Y, García-Estrada C, Balaña-Fouce R. Current status on prevention and treatment of canine leishmaniasis. Veterinary Parasitology. 2016;227:98-114. doi: 10.1016/j.vetpar.2016.07.011.

81. Reithinger R, Ceballos L, Stariolo R, Davies CR, Gürtler RE. Chagas disease control: Deltamethrin-treated collars reduce Triatoma infestans feeding success on dogs. Transactions of the Royal Society of Tropical Medicine and Hygiene. 2005;99(7):502-8. doi: 10.1016/j.trstmh.2004.11.013.

82. Reithinger R, Coleman PG, Alexander B, Vieira EP, Assis G, Davies CR. Are insecticide-impregnated dog collars a feasible alternative to dog culling as a strategy for controlling canine visceral leishmaniasis in Brazil? International Journal for Parasitology. 2004;34(1):55-62. doi: 10.1016/j.ijpara.2003.09.006. PubMed PMID: WOS:000188377300007.

83. Reithinger R, Davies CR. Canine leishmaniasis: Novel strategies for control. Trends in Parasitology. 2002;18(7):289-90. doi: 10.1016/S1471-4922(02)02296-1.

84. Reithinger R, Teodoro U, Davies CR. Topical insecticide treatments to protect dogs from sand fly vectors of leishmaniasis. Emerging Infectious Diseases. 2001;7(5):872-6. doi: 10.3201/eid0705.017516.

85. Ribas LM, Zaher VL, Shimozako HJ, Massad E. Estimating the Optimal Control of Zoonotic Visceral Leishmaniasis by the Use of a Mathematical Model. Scientific World Journal. 2013. doi: 10.1155/2013/810380. PubMed PMID: WOS:000323344400001.

86. Ribeiro RR, Michalick MSM, Da Silva ME, Dos Santos CCP, Frézard FJG, Da Silva SM. Canine Leishmaniasis: An Overview of the Current Status and Strategies for Control. BioMed Research International. 2018;2018. doi: 10.1155/2018/3296893.

87. Ribeiro VM, Rajao RA, de Araujo Diniz S, Michalick MSM. Evaluation of the potential transmission of visceral lelshmaniasis in a canine shelter. Revue De Medecine Veterinaire. 2005;156(1):20-2. PubMed PMID: WOS:000227165300004.

88. Roberts MTM. Current understandings on the immunology of leishmaniasis and recent developments in prevention and treatment. British Medical Bulletin. 2005;75-76:115-30. doi: 10.1093/bmb/ldl003. PubMed PMID: WOS:000240936000008.

89. Romero GAS, Boelaert M. Control of Visceral Leishmaniasis in Latin America-A Systematic Review. Plos Neglected Tropical Diseases. 2010;4(1). doi: 10.1371/journal.pntd.0000584. PubMed PMID: WOS:000274179500012.

90. Rosypal AC, Zajac AM, Lindsay DS. Canine visceral leishmaniasis and its emergence in the United States. Veterinary Clinics of North America-Small Animal Practice. 2003;33(4):921-+. doi: 10.1016/s0195-5616(03)00030-5. PubMed PMID: WOS:000184259900015.

91. Ruiz de Ybáñez R, del Río L, Martínez-Carrasco C, Segovia M, Cox J, Davies C, et al. Questionnaire survey on Canine Leishmaniosis in southeastern Spain. Veterinary Parasitology. 2009;164(2-4):124-33. doi: 10.1016/j.vetpar.2009.06.013.

92. Saridomichelakis MN, Koutinas AF. Cutaneous involvement in canine leishmaniosis due to Leishmania infantum (syn. L. chagasi). Veterinary Dermatology. 2014;25(2):61-+. doi: 10.1111/vde.12105. PubMed PMID: WOS:000333404900001.

93. Seva AD, Martcheva M, Tuncer N, Fontana I, Carrillo E, Moreno J, et al. Efficacies of prevention and control measures applied during an outbreak in Southwest Madrid, Spain. Plos One. 2017;12(10). doi: 10.1371/journal.pone.0186372. PubMed PMID: WOS:000412980300030.

94. Sevá AP, Ovallos FG, Amaku M, Carrillo E, Moreno J, Galati EA, et al. Canine-Based Strategies for Prevention and Control of Visceral Leishmaniasis in Brazil. PloS one. 2016;11(7):e0160058. doi: 10.1371/journal.pone.0160058.

95. Shimozako HJ, Wu J, Massad E. The Preventive Control of Zoonotic Visceral Leishmaniasis: Efficacy and Economic Evaluation. Computational and Mathematical Methods in Medicine. 2017;2017. doi: 10.1155/2017/4797051.

96. Silva RAE, Andrade AJ, Quint BB, Raffoul GES, Werneck GL, Rangel EF, et al. Effectiveness of dog collars impregnated with 4% deltamethrin in controlling visceral leishmaniasis in Lutzomyia longipalpis (Diptera: Psychodidade: Phlebotominae) populations. Memorias do Instituto Oswaldo Cruz. 2018;113(5):e170377. Epub 2018/03/29. doi: 10.1590/0074-02760170377. PubMed PMID: 29590235; PubMed Central PMCID: PMCPMC5868867.

97. Silva S, Gomes LB, Carvalho P, Santos A, Borges L, Oliveira CSF, et al. Effectiveness of the mass use of deltamethrin-impregnated dog collars for preventing transmission of canine leishmaniasis by Lutzomyia spp.: a cluster randomized controlled trial. Preventive veterinary medicine. 2019;171. doi: 10.1016/j.prevetmed.2019.104770. PubMed PMID: CN-01988564.

98. Solano-Gallego L, Fernandez-Bellon H, Serra R, Gallego M, Ramis A, Fondevila D, et al. Cutaneous leishmaniosis in three horses in Spain. Equine Veterinary Journal. 2003;35(3):320-3. doi: 10.2746/042516403776148336. PubMed PMID: WOS:000182848700019.

99. Solano-Gallego L, Koutinas A, Miro G, Cardoso L, Pennisi MG, Ferrer L, et al. Directions for the diagnosis, clinical staging, treatment and prevention of canine leishmaniosis. Veterinary Parasitology. 2009;165(1-2):1-18. doi: 10.1016/j.vetpar.2009.05.022. PubMed PMID: WOS:000271160200001.

100. Solano-Gallego L, Mirá G, Koutinas A, Cardoso L, Pennisi MG, Ferrer L, et al. LeishVet guidelines for the practical management of canine leishmaniosis. Parasites and Vectors. 2011;4(1). doi: 10.1186/1756-3305-4-86.

101. Toepp AJ, Bennett C, Scott B, Senesac R, Oleson JJ, Petersen CA. Maternal Leishmania infantum infection status has significant impact on leishmaniasis in offspring. Plos Neglected Tropical Diseases. 2019;13(2). doi: 10.1371/journal.pntd.0007058. PubMed PMID: WOS:000459970700021.

102. Travi BL, Cordeiro-da-Silva A, Dantas-Torres F, Miro G. Canine visceral leishmaniasis: Diagnosis and management of the reservoir living among us. Plos Neglected Tropical Diseases. 2018;12(1). doi: 10.1371/journal.pntd.0006082. PubMed PMID: WOS:000424022700011.

103. Trigo J, Abbehusen M, Netto EM, Nakatani M, Pedral-Sampaio G, de Jesus RS, et al. Treatment of canine visceral leishmaniasis by the vaccine Leish-111f+MPL-SE. Vaccine. 2010;28(19):3333-40. doi: 10.1016/j.vaccine.2010.02.089. PubMed PMID: WOS:000277677000011.

104. Wasserberg G, Poche R, Miller D, Chenault M, Zollner G, Rowton ED. Imidacloprid as a potential agent for the systemic control of sand flies. Journal of Vector Ecology. 2011;36:S148-S56. doi: 10.1111/j.1948-7134.2011.00125.x. PubMed PMID: WOS:000287927300020.

105. Werneck GL. Visceral leishmaniasis in Brazil: rationale and concerns related to reservoir control. Revista De Saude Publica. 2014;48(5):851-5. doi: 10.1590/s0034-8910.2014048005615. PubMed PMID: WOS:000344469200017.

106. Wylie CE, Carbonell-Antonanzas M, Aiassa E, Dhollander S, Zagmutt FJ, Brodbelt DC, et al. A systematic review of the efficacy of prophylactic control measures for naturally-occurring canine leishmaniosis, part I: Vaccinations. Preventive Veterinary Medicine. 2014;117(1):7-18. doi: 10.1016/j.prevetmed.2014.06.015. PubMed PMID: WOS:000346883000002.

107. Wylie CE, Carbonell-Antoñanzas M, Aiassa E, Dhollander S, Zagmutt FJ, Brodbelt DC, et al. A systematic review of the efficacy of prophylactic control measures for naturally occurring canine leishmaniosis. Part II: Topically applied insecticide treatments and prophylactic medications. Preventive Veterinary Medicine. 2014;117(1):19-27. doi: 10.1016/j.prevetmed.2014.06.016.

108. Xiong G, Jin C, Hong Y, Su Z, Xue P, Xie W, et al. Studies on the deltamethrin-medicated bath of domestic dogs for interrupting visceral leishmaniasis transmission. Zhongguo ji sheng chong xue yu ji sheng chong bing za zhi = Chinese journal of parasitology &amp; parasitic diseases. 1995;13(3):178-81.

109. Yaghoobi-Ershadi MR. Control of Phlebotomine Sand Flies in Iran: A Review Article. Journal of Arthropod-Borne Diseases. 2016;10(4):429-44. PubMed PMID: WOS:000386058700001.
